# The acquisition and retention of urinary catheterisation skills using surgical simulator devices: teaching method or student traits

**DOI:** 10.1186/s12909-014-0264-3

**Published:** 2014-12-21

**Authors:** Peadar S Waters, Terri McVeigh, Brian D Kelly, Gerard T Flaherty, Dara Devitt, Kevin Barry, Michael J Kerin

**Affiliations:** Discipline of Surgery, School of medicine, National University of Ireland Galway, Galway, Ireland; Department of Medicine, School of medicine, National University of Ireland Galway, Galway, Ireland

**Keywords:** Simulation, High fidelity simulators, Skill acquistion, Surgical training, Skill retention, Learning decay

## Abstract

**Background:**

The acquisition of procedural skills is an essential component of learning for medical trainees. The objective of this study was to assess which teaching method of performing urinary catheterisation is associated with most efficient procedural skill acquisition and retention. We evaluated factors affecting acquisition and retention of skills when using simulators as adjuncts to medical training.

**Methods:**

Forty-two second year medical students were taught urinary catheter insertion using different teaching methods. The interactive group (n = 19) were taught using a lecture format presentation and a high fidelity human urinary catheter simulator. They were provided with the use of simulators prior to examination. The observer group (n = 12) were taught using the same method but without with simulator use prior to examination. The didactic group (n = 11) were taught using the presentation alone. Student characteristics such as hand dexterity and IQ were measured to assess for intrinsic differences. All students were examined at four weeks to measure skill retention.

**Results:**

Catheter scores were significantly higher in the interactive group (p < 0.005). Confidence scores with catheter insertion were similar at index exam however were significantly lower in the didactic group at the retention testing (p < 0.05). Retention scores were higher in the interactive group (p < 0.001). A significant positive correlation was observed between laparoscopy scores and time to completion with overall catheter score (p < 0.05). Teaching method, spatial awareness and time to completion of laparoscopy were significantly associated with higher catheter scores at index exam (p = 0.001). Retention scores at 4 weeks were significantly associated with teaching method and original catheter score (p = 0.001).

**Conclusion:**

The importance of simulators in teaching a complex procedural skill has been highlighted. Didactic teaching method was associated with a significantly higher rate of learning decay at retention testing.

**Electronic supplementary material:**

The online version of this article (doi:10.1186/s12909-014-0264-3) contains supplementary material, which is available to authorized users.

## Background

With the introduction of the European working time directive and the regulation of the profession and training bodies, there is an increased emphasis surrounding the acquisition, assessment and retention of procedural skills within medical training facilities [1-3]. With less time to train students and junior doctors, there is a requirement for procedural training to be structured in order to improve skill translation in the least time with maximum efficiency. It is well documented in the literature that poor clinical skills and competency can compromise patient care and safety [4]. Ascertaining competence in a task is a complex, multifactorial process that takes time and experience. It is therefore imperative to provide suitable educational opportunities at an early stage of medical training to ensure competency amongst medical trainees [5].

Prior to the introduction of the Halsted’s apprenticeship model of training at the beginning of the 20th century, trainees were immersed in the hospital setting and expected to learn the relevant skills in an unstructured manner. With the advent of more complex procedures such as laparoscopy and robotics, increasing efforts have been deployed to teach fundamental skills in surgical teaching laboratories [6]. Pioneering work by the Toronto group reported that simulation based training improved translation of core surgical skills from the bench into the operating room [7]. During the introduction of laparoscopic cholecystectomy, complication rates such as common bile duct damage, bleeding and iatrogenic injuries increased by 25% [8]. It was clearly evident from various studies that complications rates could be reduced to an acceptable rate with training using inanimate simulators [9-11]. Medical students and junior doctors now undergo simulation training modules in order to learn procedural skills such as suturing and urinary catheterisation in a controlled environment free of any adverse consequences to actual patients.

Simulation based training is cost effective and benefits the health sector budget with faster procedural times, fewer complications encountered and overall greater efficiency within the hospital [12-14]. External factors in skill acquisition have been analysed such as the role of feedback to trainees, practice frequency and the type of simulators used but to date, little evidence exists on which trainees benefit most from simulation based training. In addition debate continues regarding standards of skills acquired by medical students and junior doctors and suitability of current teaching methods [15]. Despite multiple studies highlighting the advantages of learning through the use of simulators, didactic methods are still used to teach students and junior doctors various procedural skills. Through simulation, students exhibited similar stress levels to real life scenarios that would not be reproducible using lecture techniques [16]. Studies analysing confidence levels in carrying out procedural skills is limited. Furthermore there is limited data on individual learner characteristics when examining skill acquisition and retention using simulators. Some individuals acquire skills more rapidly than others; indeed, innate abilities and the speed of skill acquisition vary considerably among trainees, even in groups with similar levels of clinical experience [17].

The aims of this study was to assess which teaching method in performing urinary catheter insertion is associated with most efficient skill acquisition and longer learner retention in undergraduate medical students. Furthermore we aimed to understand human characteristics affecting acquisition and retention of skills when using simulators as adjuncts to medical training and to examine factors which are associated with “learning decay” at retention testing.

## Methods

### Teaching methods

Forty-two second year medical students at NUI Galway were randomly allocated into three groups to attend teaching sessions on urinary catheter insertion. The first 21 males and 21 females that voluntarily agreed to participate in the study were included. Consent was obtained from all participants taking part in the study. All teaching sessions and lecture format presentations were carried out by an experienced urologist with more than 5 years teaching experience. The lecture presentation was a 15 slide tutorial lasting 90 minutes outlining the indications, consent, complications and diagrammatical instructions for urinary catheter insertion in patients. The Interactive group (n = 19) were taught using the lecture presentation and a high fidelity human urinary catheter simulator All students within this group were provided with simulators to facilitate practice prior to examination for 30 minutes. The Observer group (n = 12) were taught using the same presentation and observed the urologist carrying out catheter insertion on the simulator for 30 minutes, however they were not provided with hands on experience prior to examination. The Didactic group (n = 11) were taught using lecture format presentation alone and had no access to the simulator. Exclusion criteria included previous experience with urethral catheterisation. All students were asked to complete questionnaires measuring their confidence with catheterisation at different stages of the teaching process along with individual characteristics such as hobbies, examination results, type of learning style as outlined by Honey and Mumford and career motivation which may account for differences in skill acquisition and retention [18]. Students were informed that answers to questionnaires and results were annonomised to prevent response bias.

### Observed structured clinical examination

Within 2 hours of each teaching session the students underwent an Observed Structured Clinical Examination (OSCE). Students were examined using 4 stations at which examinations ran for 6 minutes each. They were graded by two observers using a standardised marking sheet to prevent bias (Additional file 1: Appendix 1). The examiners were blinded to the teaching method each group received. Instructions for each station prior to entry were available to read.

Outcomes measured at the individual stations were:

1. Patient consenting station – Assessing understanding of the procedure.
2. Catheter Insertion Score – Assessing knowledge and synthesis of the procedure. Each participant performed the task of urinary catheterisation using Advanced Human Patient Simulators (AHPS, Figure 1). The AHPS are fitted with urinary bags containing yellow liquid. The presence of urine backflow into the catheter with safe balloon inflation using an aseptic technique throughout represented completion of the task.

**Figure 1:**
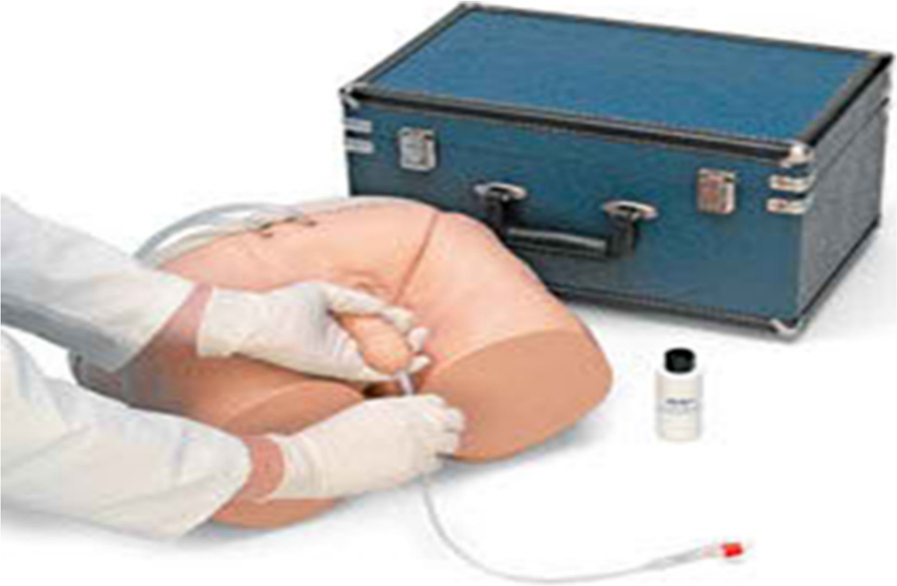
**Advanced Human Patient Simulators (AHPS) - male urinary catheter model.**
3. Intellectual Quotient (IQ) derived from the standardised MENSA question bank assessing spatial awareness, memory testing, concentration, and subgroup classification [19].
4. Hand dexterity was measured using a beginner’s virtual reality laparoscopy exercise on the ProMis Laparoscopy trainer® (Figure 2). The task measured accuracy of pathway, economy of movement and time to task completion. Participants were asked to move 3 bean shaped objects from one tray to the next. They were also asked to place a rubber ring on to a hook. This instrument measured time taken to perform each procedure, excessive movement of the instrument tip (hand dexterity) and overall pathway accuracy of instrument (hand eye co-ordination).

**Figure 2:**
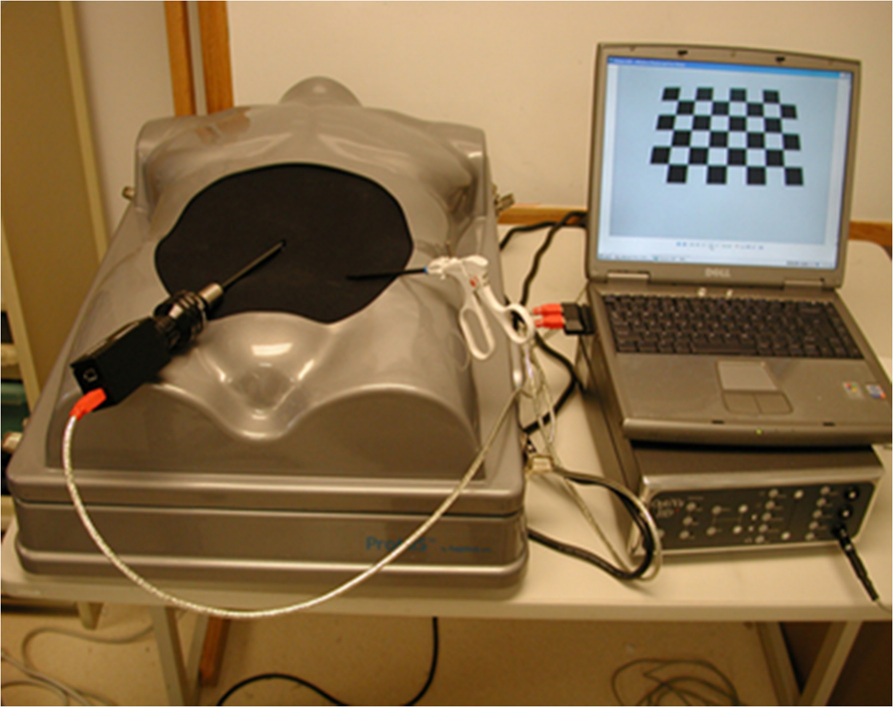
**ProMis laparoscopy trainer®.**

All students were re-examined at 4 weeks to measure retention of catheterisation skills. Students were requested not to up-skill in the interim.

### Ethics

Ethical approval was obtained from the Galway University Hospitals ethics committee, following submission of the standard ethics form and chairperson’s review.

### Data collection and analysis

Data was collected and analysed using Minitab version 16. Analysis of Variance (ANOVA) was conducted to examine the difference between group means. Regression analysis was carried out to determine the relationship between variables. A *p*-value of < 0.05 was considered significant.

## Results

This study comprised of 42 second year medical students from National University of Ireland Galway. The average age was 20.95 years with 21 males and 21 female participants, respectively. Twenty nine students were interested in pursuing a surgical career.

### Student confidence levels with urinary catheter insertion

**Figure 3:**
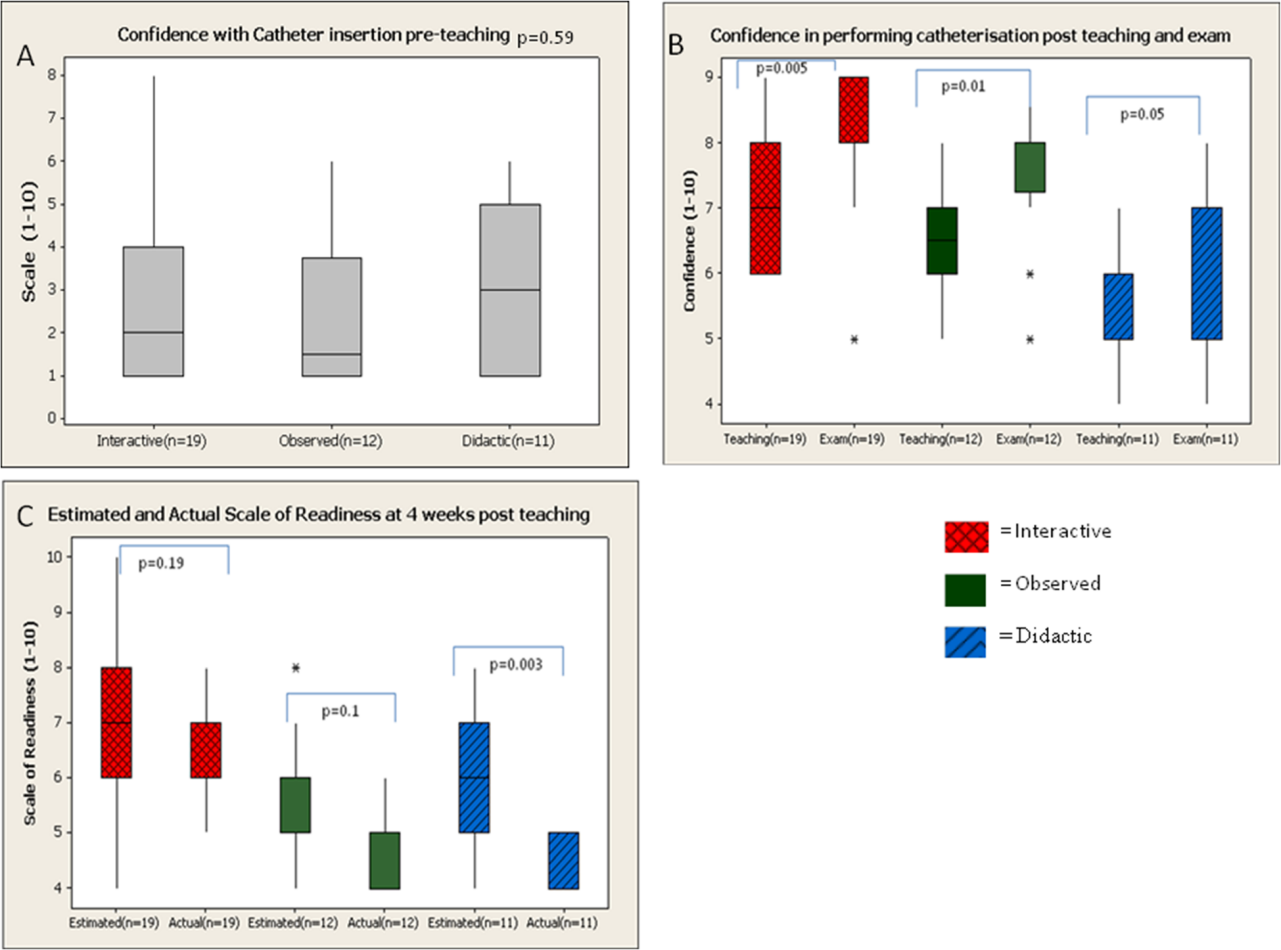
**Confidence with catheter insertion prior to teaching.** **A**: There was no significant difference observed in confidence levels for all groups prior to teaching (p = 0.59). **B**: Confidence of students in performing catheterisation after teaching and OSCE examination: There was a significant increase in confidence amongst students with urinary catheter insertion after teaching and examination session. **C**: Estimated and actual confidence levels of urinary catheterisation at the 4 week retention test: There was a significant decrease in estimated confidence levels for catheter insertion at the four week retention test measured at index exam and the actual confidence levels at the four week retention test with student thought by the didactic method (p = 0.003).

All students were asked to document their level of confidence with catheterisation at different stages of the teaching and examination process. Prior to teaching there was no significant difference in the confidence of students in performing urinary catheter insertion (*p* = 0.59, Figure 3a). On a scale of 1–10 the average score was 2.64 ± 1.54 for the interactive group, 1.6 ± 1.45 in the observed group and 2.66 ± 1.62 in the didactic group. As expected there was a significant increase in confidence in student ability for catheter insertion post teaching despite teaching method (*p* < 0.05). Student confidence levels in performing catheterisation were found to be significantly higher for all teaching groups after they had undergone the examination process (7.4 ± 0.96 v 8.1 ± 0.68, *p* = 0.005, 6.5 ± 1.03 v 7.54 ± 1.07, p = 0.01 and 5.4 ± 0.79 v 6 ± 1.12 p < 0.05, Figure 3b respectively). The largest increase in confidence levels was observed in the interactive group followed by the observed group, (*p* = 0.005, 0.01) respectively.

All students were asked to document what they estimated their level of confidence would be with urinary catheter insertion 4 weeks after their index examination. Prior to undergoing retention testing, actual confidence levels in performing catheter insertion were measured again. There was no significant difference observed (*p* > 0.05) in estimated and actual confidence level with catheter insertion for both the interactive group and observed group (6.83 ± 1.27 v 6.57 ± 1.07 p = 0.19, 5.44 ± 1.34 v 4.94 ± 1.09 p = 0.1 respectively, Figure 3c). However within the didactic group, the level of estimated confidence was significantly overestimated at the time of index examination compared to actual level of confidence measured at retention testing 4 weeks later (6.1 ± 1.07 v 4.53 ± 0.51, p < 0.005).

### Catheterisation scores at index exam and four week retention test

**Figure 4:**
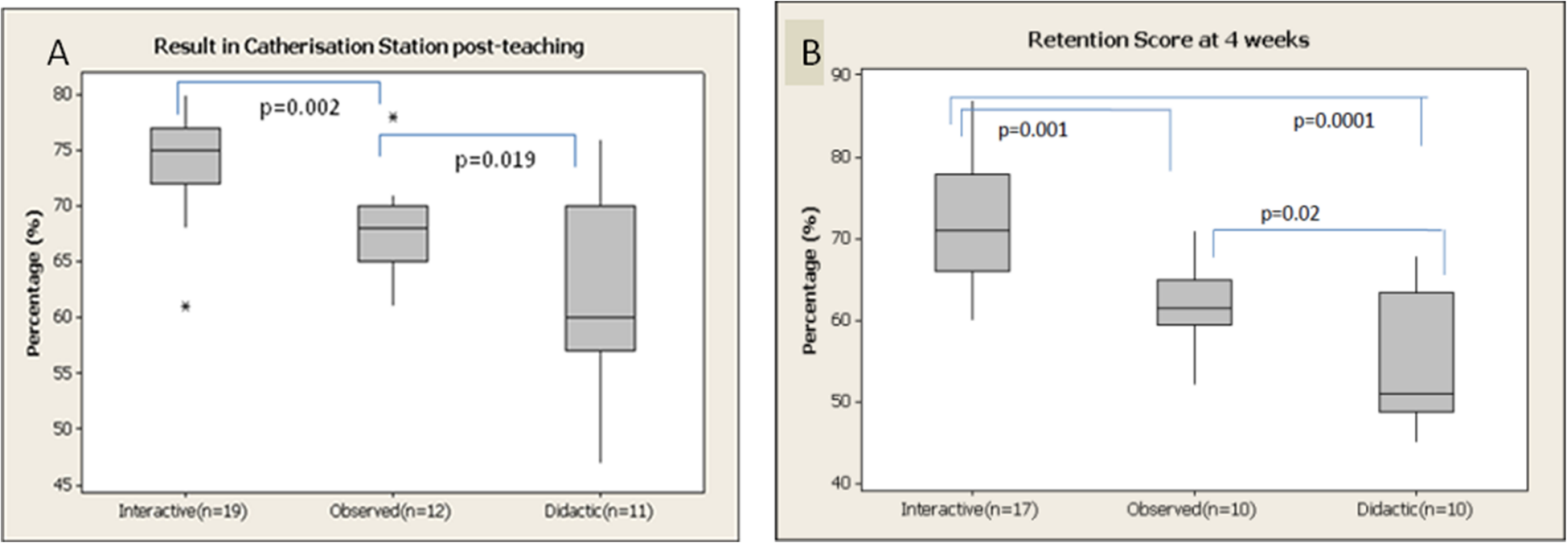
**Examination scores of each group post teaching using three different teaching methods. A**: The interactive group scored highest at index exam followed by the observed group. The didactic group scored significantly less (p = 0.019). **B**: Retention Scores at 4 weeks: At the 4 week retention test the interactive group scored significantly higher followed by the observed group. The didactic group scored significantly less.

Index examination scores for urinary catheter insertion were measured within 1 hour of each student undergoing a teaching session. The interactive group (n = 19) had significantly higher scores at index examination compared to the observed (n = 12) and didactic (n = 11) teaching groups (74% ±6.09%, 68% ±3.84%, 60% ±9.14%, respectively p = 0.002 Figure 4a). The didactic group had significantly lower scores compared to the other two teaching methods (p = 0.0001 and 0.02, respectively). There was also greater variation in scores observed in the didactic group. There was no significant difference observed between each teaching group and results achieved at the consenting station. However, while students within the interactive group generally scored higher, scores were not found to be statistically significant (*p* = 0.17, 0.57).

Retention scores for urinary catheter insertion were measured at 4 weeks post initial teaching. Students taught using the interactive simulation method scored significantly higher at their retention test than the other teaching methods (71% ±8.9%, 62% ±5.84% 51% ± 6.21% respectively, *p* = 0.001, Figure 4b). Students taught by the observed group also scored significantly higher than the didactic group (*p* = 0.02).

### Factors associated with higher catheter insertion scores at index exam and four week retention test

**Figure 5:**
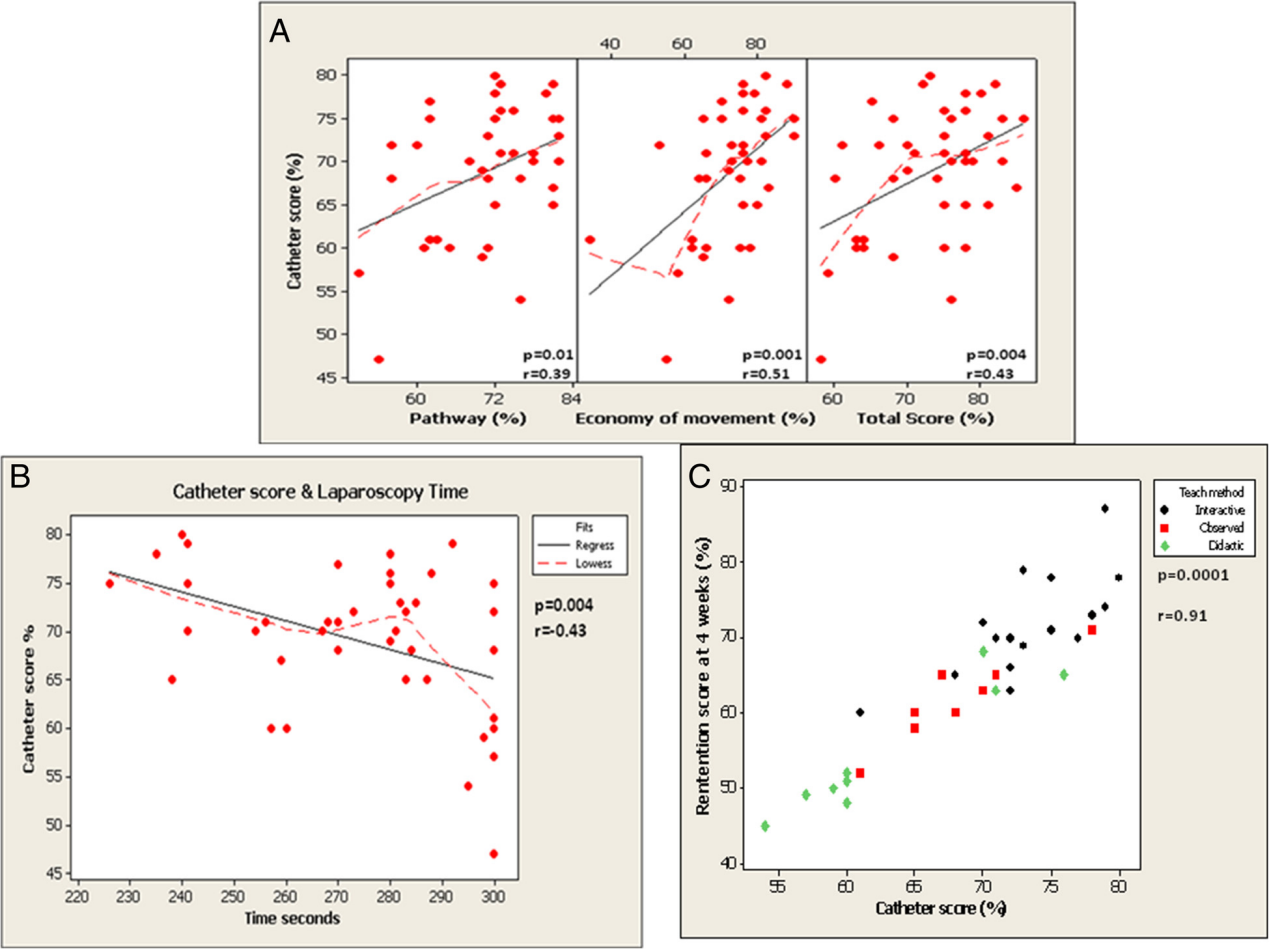
**Factors affecting catheter scores at index exam. A**: Correlation analysis exhibits a strong positive relationship between overall catheter scores at index examination and pathway efficiency, economy of movement and total laparoscopic score. **B**: Factors affecting catheter scores at index exam: A Significant Negative Correlation observed between catheter score and time to completion of laparoscopy task. **C**: A significant correlation between initial catheter scores at index exam and those at the retention test: A significant positive correlation was also observed between catheter score at index examination and scores at the retention test four weeks later. This correlation remained significant within each teaching group.

All forty-two students underwent a beginner’s laparoscopic training drill as part of their examination process. Its function was to measure individual hand dexterity by assessing economy of movement and pathway efficiency with laparoscopic instruments. Time to task completion and overall laparoscopic score for the training drill was also measured. A significant correlation was observed between pathway efficiency, economy of movement and total laparoscopy score with overall catheter scores at index examination (*p* = 0.01, 0.001, 0.004, r = 0.39, 0.51, 0.43) respectively (Figure 5a).

A significant negative correlation was also observed between time to completion of the laparoscopic task and overall catheter insertion score measured at index examination (Figure 5b). Less time required to complete the laparoscopic task was associated with higher catheter insertion scores at index exam. A significant positive correlation was also observed between catheter score at index examination and scores at the retention test four weeks later (p = 0.0001, r = 0.91, Figure 5c). This correlation remained significant within each teaching group.

A multivariate analysis was carried out to assess the relationship between factors associated with scores for catheter insertion at index examination and at the retention test four weeks after teaching. Teaching method, time to completion of laparoscopic task and spatial awareness measured at IQ testing were significantly associated with catheter scores (*p* = 0.000, 0.039, 0.018) respectively.

A multivariate analysis was carried out to identify the factors associated with higher catheter scores at the retention test. Teaching method and catheter score at index examination were found to be significantly associated with catheter scores at retention testing (*p* = 0.001, 0.000). There was also a trend towards significance noted for those students who played music (*p* = 0.058).

## Discussion

The role of simulation is to recreate a clinical scenario that is representative of a true life situation. Multiple studies have shown that skills learned at the bench using simulators are translated into the operating room [20]. This allows trainees to focus more on operative strategy and managing operative complications rather than wasting valuable and expensive operating room time on the initial refinement of psychomotor skills [21].

In this study the degree of readiness or confidence levels of individual students with catheter insertion was measured during different time points. There was no significant difference in confidence levels for each group prior to teaching. Confidence levels were found to be significantly lower in the didactic group after their initial teaching session. Interestingly however, confidence levels were significantly highest for all students after undergoing an OSCE format examination for catheter insertion. This outlines the importance of the examination process in assuring students of their ability to perform a procedural skill competently. A limitation to this study is that although all students underwent the OSCE examination process, it is difficult to assess whether this increase in confidence may simply be due to more hands on experience rather than the examination process itself. Assessments have been shown to promote reflective practice [22]. This helps students identify gaps in their knowledge and skill set in carrying out certain tasks. Moreover it promotes self learning and allows students to develop higher levels of cognition [23]. Through simulation, trainees apply their knowledge to create or synthesise a solution. The importance of using a simulator was evident from our study with the didactic group reporting significantly higher levels of confidence with catheter insertion after using the simulator once during their examination. Despite this however, the same group significantly overestimated their confidence in catheter insertion at index examination compared to actual confidence measured four weeks later by retention testing. This overestimation was not experienced by the interactive or observed groups. A study examining confidence in performing on real life patients after simulation training showed that simulation-trained residents had higher levels of confidence and performed better than untrained controls during the initial stages of training, after which there was no difference [24]. This finding indicates that the learning curve which is commonly encountered when performing a new technique could be reduced by performing simulation based training. The use of simulators by students prior to examination is associated with significantly higher scores at index examination than those just simply observing the process. This highlights the benefit of “skill reinforcement”. In a study analysing the acquisition of laparoscopic skills amongst junior trainees, trainees who underwent simulation based training were never outperformed by the non-simulator group throughout all parameters observed [25].

In the current study, significant differences in performance were also observed at the four week retention test. The interactive group significantly outperformed the observed and didactic groups. The retention of skills in medical practice is critical. A limitation acknowledged in this current study was the relatively short duration from index exam to retention testing, small study group and lack of randomisation. Although well documented in the literature that proficiency based progression training has been qualitatively shown to the optimal approach to skill acquisition, due to time constraints it was no possible to analyse this within our cohort [26]. Retention testing at four weeks was chosen as students were simultaneously undergoing end of year examinations. This raises the issue of the appropriate time to carry out retention testing in order to establish the long term durability of learning through simulation. One study examining skill retention in basic life support (BLS) amongst specialist registered cardiac nurses found that skill degradation occurred as early as 1 – 3 months after training [27]. Similar findings to our study were observed in a prospective study analysing the use of high fidelity simulation compared to didactic teaching in performing airway intubation. Those taught by simulation were seen to outperform those taught by lectures and this improved performance was also found to be statistically significant at 4 months [28].

Skill degradation is a serious issue in medical education and is associated with increased procedural times, costs and complications [29-32]. Factors considered important for skill retention include the duration of retention interval, the quality of the original training, task complexity, and intrinsic learner differences [33]. Studies to date have analysed the impact of extrinsic factors such as practice distribution, task complexity and feedback on motor skill acquisition and retention [34]. The importance of feedback during skill acquisition has been highlighted in multiple studies surrounding the recent introduction of Hybrid Simulation [35-37]. Although this current study utilises an In-Vitro method of simulation teaching it allows the examination and impact of intrinsic learner differences. We observed that regardless of the students learning style, ethnicity and hand dominance, there was no significance difference in skill retention and acquisition. Furthermore with 69% of students highlighting their interest in pursuing a career in surgery, career motivation did not impact on overall performance.

We found that students who scored higher in spatial awareness and hand dexterity tasks such as time to laparoscopic task completion, economy of movement and instrument pathway scored higher in catheter insertion at index exam. Moreover there was a significant correlation between scores achieved by individual students at initial testing and those at four weeks highlighting that some students possess innate skills. This is easy to understand as it has been long recognised that some individuals master a certain skill set such as laparoscopy in a relatively short period while others struggle to learn, take longer and may never reach competency. Interestingly, computer gaming and playing sports did not impact on performance in this study. Previous studies have shown significant association with certain pre-learned skills such as computer gaming, music and sport with easier acquisition of new procedural skills [38-40]. It is important to recognise that this is an association and does not imply causality. The ability to play a musical instrument did not impact on catheter score at index exam although there was a trend towards significance in impacting on retention scores which may have become statistically significant within a larger cohort. Finally the type of teaching method significantly impacted on performance at index examination and retention. In a multivariate analysis examining both student and teaching factors, the use of simulators within the teaching method was the single significant factor influencing catheter insertion scores both at index examination and retention testing.

## Conclusions

The importance of simulators in teaching a complex procedural skill has been highlighted. Teaching and allowing students to practice their skills on simulators is associated with higher index and retention examination scores. This study also highlights the importance of the examination process during teaching in assuring students of their ability to carry out procedural skills. Simulator training has been shown to suit all types of individuals regardless of learning style. Students with increased manual dexterity and spatial awareness score significantly higher with the use of simulators in teaching. Skill degradation or “decay” was significantly higher in the didactic teaching group. In order to optimise training in procedural skills for the future there needs to be an increase in the provision of simulation based training. This will allow for a more efficient and smoother translation of procedural skills from the non clinical environment of the skills laboratory to the hospital wards and operating room.

## Additional file

Additional file 1:
**Appendix 1.** Standardised OSCE marking sheet for urinary catheter insertion.
