# Supplementary material for: The acquisition and retention of urinary catheterisation skills using surgical simulator devices: teaching method or student traits
Source: BMC Med Educ. 2014 Dec 21;14:264. doi: 10.1186/s12909-014-0264-3 (PMC4323138; doi:10.1186/s12909-014-0264-3)
Supplement: Additional file 1: — Appendix 1. Standardised OSCE marking sheet for urinary catheter insertion. [file 12909_2014_264_MOESM1_ESM.doc]

**URINARY CATHETERISATION CANDIDATE ID:**

| **Task** | **Competent performance** | **Inadequate performance** |
| --- | --- | --- |
| **Performs hand hygiene** |  |  |
| **Cleans trolley** |  |  |
| **Selects correct equipment** |  |  |
| **Places equipment underneath clean trolley** |  |  |
| **Performs hand hygiene at bedside** |  |  |
| **Consents and discloses to patient** |  |  |
| **Puts on apron** |  |  |
| **Sets up the sterile field and trolley** |  |  |
| **Positions and exposes patient** |  |  |
| **Indicates that he would wash his hands** |  |  |
| **Puts on sterile gloves correctly** |  |  |
| **Applies drape to patient** |  |  |
| **Cleans penis with saline** |  |  |
| **Changes sterile gloves (optional step if doing 2 handed technique)** |  |  |
| **Uses gauze swab to hold penis** |  |  |
| **Administers local anaesthetic** |  |  |
| **Indicates that he would allow 5 minutes for anaesthetic gel to work** |  |  |
| **Places tray under penis** |  |  |
| **Opens catheter packaging** |  |  |
| **Inserts catheter correctly** |  |  |
| **Obtains water in the catheter/kidney dish** |  |  |
| **Connects the urinary collection bag** |  |  |
| **Indicates he would inflate the balloon after water appears in bag/dish** |  |  |
| **Replaces foreskin** |  |  |
| **Dries patient/simulator** |  |  |
|  |  |  |
| **Disposes of waste into yellow bag** |  |  |
| **Removes gloves and indicates that he would wash hands** |  |  |
| **Indicates that he would document procedure in patient’s notes** |  |  |
|  |  |  |
| **Overall ability** | Meets standard | Below standard |
| **Automatic fail if red requirement omitted or inadequately performed** |  |  |

**Examiner’s signature..............................................**
